# p8 deficiency leads to elevated pancreatic beta cell mass but does not contribute to insulin resistance in mice fed with high-fat diet

**DOI:** 10.1371/journal.pone.0201159

**Published:** 2018-07-24

**Authors:** Marcus Hollenbach, Nora Klöting, Ines Sommerer, Jana Lorenz, Mario Heindl, Matthias Kern, Joachim Mössner, Matthias Blüher, Albrecht Hoffmeister

**Affiliations:** 1 Department of Medicine, Neurology and Dermatology, Division of Gastroenterology and Rheumatology, University of Leipzig, Leipzig, Germany; 2 IFB Adiposity Disease, University of Leipzig, Leipzig, Germany; 3 German Diabetes Center Leipzig, University of Leipzig, Leipzig, Germany; 4 Department of Medicine, Neurology and Dermatology, Division of Endocrinology and Nephrology, University of Leipzig, Leipzig, Germany; University of Szeged, HUNGARY

## Abstract

**Background:**

p8 was initially described as being overexpressed in acute pancreatitis and encoding a ubiquitous stress protein. Analysis of insulin sensitivity and glucose tolerance in p8-knockout and haplodeficient mice revealed counterintuitive results. Thus, we determined glycemic control of p8 in mice fed with standard (SD) and high-fat diet (HFD).

**Methods:**

p8^-/-^ and wild type (p8^+/+^) mice were used for analysis of glucagon (immunohistochemistry), insulin levels (ELISA) and beta cell mass. Hyperinsulinemic- euglycemic glucose clamp technique, i.p. glucose tolerance test (ipGTT), i.p. insulin tolerance test (ipITT) and metabolic chamber analysis were performed in SD (4% fat) and HFD (55% fat) groups.

**Results:**

p8^-/-^ mice showed no differences in glucagon or insulin content but higher insulin secretion from pancreatic islets upon glucose stimulation. p8 deficiency resulted in elevated beta cell mass but was not associated with increased insulin resistance in ipGTT or ipITT. Glucose clamp tests also revealed no evidence of association of p8 deficiency with insulin resistance. Metabolic chamber analysis showed equal energy expenditure in p8^-/-^ mice and wild type animals.

**Conclusion:**

p8 depletion may contribute to glucose metabolism via stress-induced insulin production and elevated beta cell mass. Nevertheless, p8 knockout showed no impact on insulin resistance in SD and HFD-fed mice.

## Introduction

Nuclear Protein 1, Transcriptional Regulator gene (NUPR1, p8; OMIM: 614812) encodes a ubiquitous nuclear and cytoplasmic stress-activated protein and shares structural similarities with high-mobility group box proteins [1]. Additional studies indicate interaction of p8 with transcriptional cofactors including p300 and PTIP [2], as well as binding of p8 to DNA upon phosphorylation [3]. p8 has been shown to be implicated in oxidative stress via regulation of anti-oxidative enzyme heme-oxigenase-1 (HO-1) [4], and p8 is overexpressed in sepsis and pancreatitis [5,6]. Moreover, p8^-/-^ mice reveal enhanced mortality in sepsis and liver injury [7,8]. Cerulein-induced pancreatitis is ameliorated by stimulation of p8-induced pancreatitis-associated protein I (PAP I), resulting in NF-κB inactivation [9]. p8 also affects exocrine tissue regeneration after pancreatectomy and induces proliferation of islet beta cells [10].

Furthermore, p8 shows anti-apoptotic effects through inhibition of apoptosis initiator caspase 9 and effector caspases 3/7 [11]. Recently, the role of p8 has also been extensively studied in the context of cancer. Genetic knockdown of p8 was accompanied by reduced proliferation of liver cancer, glioblastoma and myeloma cells. In addition, p8 is important to maintain autolysosomal efflux of cancer cells [12–17].

Since p8 is involved in cellular mechanisms of oxidative stress and inflammation, its role in development of insulin resistance and hyperglycemia has been analyzed. Glucose and pro-inflammatory cytokines (e.g. TNF-α) lead to upregulation of p8 [9], and glucose-dependent stimulation of p8 was observed in INS-1 insulinoma cells [18]. Overexpression of p8 in human islets leads to augmented insulin secretion, as well as cellular insulin content and improved glycemic control [19]. In contrast, p8^−/−^ mice show mild insulin resistance compared to wild type animals but maintained normoglycemia through increase of beta cell mass and consecutive hyperinsulinemia [20]. On the other hand, p8 haplodeficiency decreases visceral fat deposition and ameliorates insulin resistance through upregulation of stress-induced protein hsp70 [21].

To date, the specific role of p8 in glucose homeostasis and insulin resistance remains inconclusive regarding the counterintuitive results mentioned above. Thus, we investigated p8^-/-^ mice and their wild type littermates under standard and high-fat diet conditions with examination techniques not previously used in this context. The aims of our study were (I) to determine insulin and glucagon levels from isolated pancreatic islets as well as beta cell mass in p8-knockout and wild type mice, (II) to analyze the impact of p8 on insulin resistance by means of glucose tolerance tests (ipGTT) and insulin tolerance tests (ipITT), and (III) to evaluate the effect of p8 silencing on respiratory exchange rate, physical activity and energy consumption in a metabolic chamber. For the first time in this context, we used the hyperinsulinemic-euglycemic clamp test as the gold standard test [22] to assess p8-mediated insulin resistance.

## Material and methods

### Animal studies

p8^-/-^ mice on C57BL/J6 background were a kind gift from Prof. Juan Iovanna [8,10]. The Regional Governmental Principles of Leipzig for the care and use of animals were followed and currently approved for all procedures involving animals (animal research proposal TVV 14/08; Ethical Committee at the Medical Faculty, Leipzig University; IORG0001320, IRB00001750, chairwoman: Prof. Dr. Dr. Ortrun Riha, Käthe-Kollwitz-Str. 82, D-04109 Leipzig). Animals were kept conventionally in type-II cages (four animals per cage) with dry sluice according to guideline 86/609/EWG of the European Union and local animal protection authorities. Mice were housed in pathogen-free facilities (three to five mice per group and cage) at 22 ± 2°C on a 12-h light/dark cycle. Animals were bred and kept in the animal laboratories at the University of Leipzig. Animal housing premises comprise of electronic admission control, automated air conditioner and filtering. Standard local hygienic management was used according to “Hygiene Monitoring of Mice and Rats in Various Housing Systems“, recommended by GV-SOLAS.

All mice had free access to water at all times and were fed a standard chow (4% energy from fat, Altromin Spezialfutter, Lage, Germany, SD). A subgroup of twelve knockout (p8^−/−^) and twelve wild-type mice (equal number of male and female animals) were kept on a high-fat diet (HFD) containing 55 kJ% of calories from fat (Altromin) for 20–30 wk. Food was only withdrawn if required for an experiment.

During HFD and SD, mice were observed for typical symptoms, e.g. alterations of body weight, coat, behavior (apathy) and food intake. Additionally, a score by GV-SOLAS was calculated and the feeding experiment subsequently stopped if the score reached 20 or more points, followed by euthanasia with cervical dislocation.

### Intraperitoneal insulin tolerance tests (ipITTs) and glucose tolerance tests (ipGTTs)

ipITT and ipGTT were performed in p8-knockout and wild type animals under chow and HFD diet conditions at an age of 20 wk, as previously described [23]. ipGTT was performed after an overnight fast of 14h by injecting 2g glucose per kg body weight into p8^−/−^ and littermate controls. Measurements of the blood glucose levels were taken after tail vein incision at 0 (baseline), 15, 30, 60 and 120 min after injection. ipITT was performed in random-fed animals by injecting 0.75 units/kg body weight human regular insulin (40 units Insuman Rapid; Sanofi, Frankfurt/Main, Germany). Glucose levels were determined in blood collected from tail tip immediately before and 15, 30, and 60 min after the intraperitoneal injection.

### Hyperinsulinemic-euglycemic clamp studies

After anesthesia (Isofluran, Baxter, Unterschleißheim, Germany), catheters (MicroRenathane^®^ tubing, MRE 025; Braintree Scientific Inc., Braintree, MA) were implanted in the left jugular vein and hyperinsulinemic-euglycemic clamps of 12 animals (6 male, 6 female) of each genotype were performed at the age of 30 weeks as previously described [24–27]. Briefly, the insulin clamp was conducted with a continuous infusion of human insulin at a rate of 20 mU/kg/min to lower plasma glucose levels within a physiological range (about 5 mmol/l). Physiological blood glucose concentrations were maintained by adjusting infusion of a 20% glucose solution. Steady state was ascertained when glucose measurements were constant for 20 min at a fixed glucose infusion rate (GIR) and was achieved within 120–240 min. Steady state was maintained for 45 min and blood samples (10 μL) were taken at 0 and 5 min, and then at 10-min intervals after reaching steady state. All infusions were done using micro-dialysis pumps (TSE Systems, Chesterfield, MO, USA).

### Energy expenditure in metabolic chamber

Mice (20 wk old) fed with SD and HFD (12 per group, 6 male, 6 female) were kept in a metabolic chamber (CaloSys V2.1; TSE Systems, Bad Homburg, Germany) for 72h as previously described [28,29]. During the observation period, flow of O_2_ / CO_2_ and temperature were determined. Respiratory exchange rate (RER) was calculated by means of O_2_- and CO_2_-flow measurement. Indirect calorimetry was assessed by a calorimetry module (CaloSys V2.1; TSE Systems, Bad Homburg, Germany). After 2h of acclimatization, mean oxygen consumption (VO_2_), weight change, water and food intake as well as ability to run on a treadmill, were recorded for 72h, as previously described [24]. Data were analyzed by the manufacturer’s software.

### Immunohistochemistry and morphometric analysis

Mice were sacrificed at an age of 20 wk by bleeding and cervical dislocation (12 per group, 6 male, 6 female). The pancreas was immediately removed, weighed and organ mass was related to whole body weight to obtain relative organ weights. Explanted mouse pancreata were fixed in formaldehyde solution (4%, OttoFischerGmbH, Saarbruecken, Germany) overnight, embedded in paraffin and cut into 5 μm thick longitudinal sections. One section was mounted on one slide. We analyzed six slides per pancreas (each tenth), which were equally distributed over the organ. Sections were dewaxed and rehydrated by xylol and a descending ethanol series. Subsequently, slides were boiled in target retrieval solution (pH 9, Dako, Hamburg, Germany) and cooled down for 15 min, followed by washing in PBST (Biochrom Berlin, Germany) thrice. Quenching of endogenous peroxidase-activity by incubation for 10 min with 3% H_2_O_2_ (Sigma-Aldrich) was performed twice, followed by another washing step. Sections were blocked with 5% donkey-serum (= host of secondary antibody, Sigma) for 30 min at room temperature (RT) and incubated with primary antibodies (AB) for insulin (C27C9) or glucagon (D16G10, both rabbit monoclonal AB, Cell Signaling, Cambridge, UK) for 60 min at RT. Slides were washed thrice and incubated with secondary AB (711-035-152, donkey anti-rabbit polyclonal AB, Dianova, Hamburg, Germany) for 30 min at RT. Staining was performed with DAB Peroxidase Substrate Kit (Liquid DAB+Substrate Chromogen System, Dako), following instructions of the manufacturer. Slides lacking the primary antibody were used as controls. Sections were counterstained with ready-to-use hematoxylin (Hollborn, Leipzig, Germany). Additional overview images were stained with hematoxylin (Hollborn) and eosin (0.2%, Medite, Burgdorf, Germany; HE). Stained sections were photographed with a Keyence Biozero BZ 8000 microscope. Staining intensity of DAB was quantified using a scoring system from negative to 6x positive. Quantifications of islets, area and beta cell mass (mg per pancreas) was calculated by multiplying relative DAB-positive area (the percentage of glucagon positive area over total pancreas area) by pancreas weight, as previously reported [30] using ImageJ software (ImageJ 1.45; http://rsbweb.nih.gov/ij/download.html).

### Statistical analysis

Results are expressed as mean ± standard deviation (S.D.). At least 12 animals per group (6 male, 6 female) were used for statistical analysis of each experimental setting. Unless otherwise indicated, results of male and female animals are presented. If not further specified, subgroup analysis found no differences between the male and female groups. For comparison of only two groups (metabolic chamber, pancreas size, islet size and area under the curve (AUC)), the Mann-Whitney-U-test was performed because several groups did not pass normality tests. For grouped analysis (glucagon and insulin test, ipITT and ipGTT), the two-way ANOVA with Bonferroni post test was used. Statistical analysis of clamp tests was calculated by means of one-way ANOVA with Bonferroni post-test. P values <0.05 were considered statistically significant. GraphPad Prism 4.0 software was used for calculation and drawing of graphs.

The calculation of the sample size was performed as followed: the alpha error was set to 5% and the beta error to 80%. These data and the estimation of variance were calculated by means of sample size estimation software (http://www.psycho.uni-duesseldorf.de/abteilungen/aap/gpower3).

Further supporting information can be found in supplemental material and methods.

## Results

Previous studies by Barbosa-Sampaio et al. examined whether depletion of the p8 gene produced a metabolic phenotype. No significant differences in serum glucose and insulin levels were observed in SD, and only slightly higher insulin levels were found in p8^-/-^ HFD group suggesting a questionable role of p8 on glucose homeostasis [20].

In our study, male and female p8^−/−^ mice on a C57BL/6 background showed similar body weights compared with age-matched p8^+/+^ mice (male: 25.9±2.7 g vs. 28.7±1.5 g, p>0.05; female: 20.3±1.3 g vs. 19.3±1.6 g, p>0.05).

### p8 deficiency leads to a higher number of islets and elevates beta cell mass

**Fig 1:**
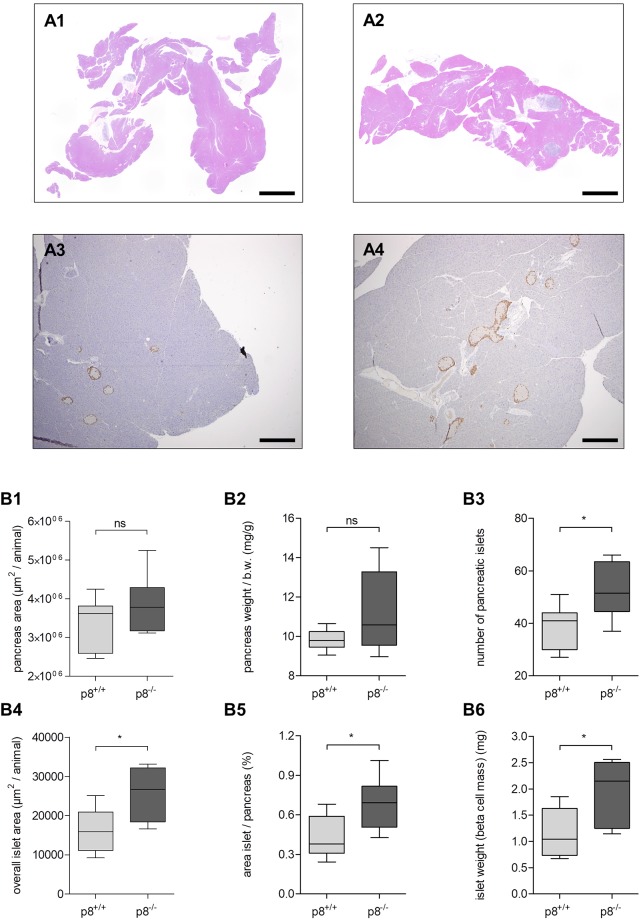
Morphologic analysis of pancreata and islets in p8^-/-^ and p8^+/+^ mice. **A1-A4**, representative HE stained bright field image of stacked whole pancreata from wild type **(A1)** and p8-knockout animals **(A2)**. Glucagon-DAB-staining indicated more islets in p8^-/-^ **(A4)** compared to p8^+/+^ animals **(A3)**. **B1-B6**, morphologic analysis of beta islets and pancreas from p8^-/-^ mice and wild type littermates. p8-knockout animals showed statistically significant higher number of islets **(B3)**, islet area **(B4, B5)**, and beta cell mass **(B6)**. No significant differences were found in mean islet size (not shown), pancreas area **(B1)** and pancreas weight **(B2)**. Results are expressed as mean ± S.D. of islets and pancreata from 12 animals (6 male, 6 female) per group. Scale bars: 1000μm (A1-A2), 100μm (A3-A4); * P<0.05.

The total number of pancreatic islets and islet size were significantly different when comparing p8-knockout and wild type mice (Fig 1). Representative images of stacked whole pancreata from both groups (Fig 1A1 and 1A2) and glucagon stained islets (Fig 1A3 and 1A4) indicated higher number of pancreatic islets in p8-knockout mice. p8 deficient mice had more islets per slide than littermate controls (53.0±10.4 vs. 38.1±8.7, p<0.05, Fig 1B3), and the total area of p8^−/−^ islets was significantly elevated (Fig 1B4). The relation of islet area to pancreas area was also higher in p8-silenced mice compared to controls (Fig 1B5). In addition, islet weight (beta cell mass) reached 1.98±0.59 mg in p8-knockout animals compared to 1.15±0.48 mg (p<0.05, Fig 1B6) in wild type mice. In contrast, mean islet size (436.4±103.5 vs. 440.0±165.1 μm^2^, p>0.05, data not shown in graph), pancreas area (Fig 1B1) and pancreas weight (Fig 1B2) did not differ significantly between both groups.

### Islets from p8^-/-^ mice secrete more insulin than wild type islets after glucose stimulation

**Fig 2:**
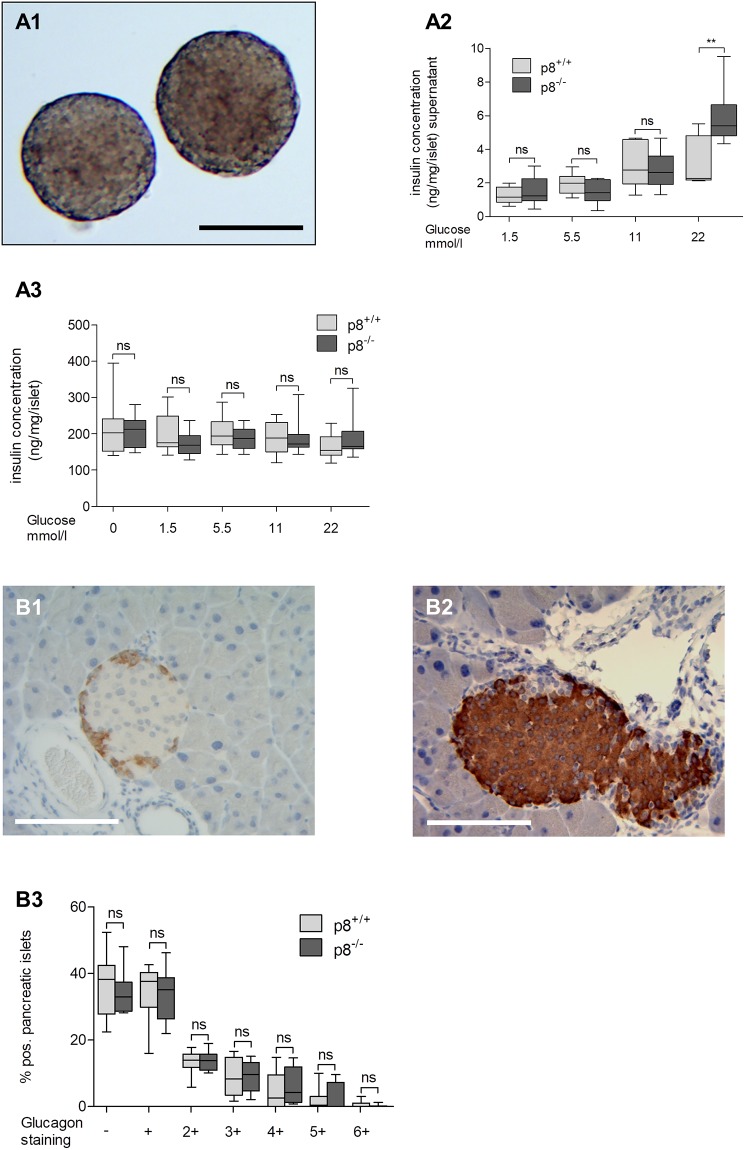
Glucagon staining intensity and insulin levels in pancreatic islet cells. **A1-A3**, insulin levels in supernatants and lysates of isolated pancreatic islets after glucose stimulation. **A1**, bright field image of isolated islets. Insulin levels were measured via ELISA after incubation of isolated islets for 2h. In protein lysates of islets **(A3)**, no statistically significant differences in insulin levels were determined upon glucose stimulation. In contrast, islet supernatants showed significant elevation of insulin after stimulation with 22 mM glucose in p8-knockout islets **(A2)**. **B1-B2**, representative images of DAB stained pancreatic slides for glucagon **(B1)** and insulin **(B2)** indicating pancreatic islets. Slides were counterstained with hematoxylin. Quantifications of DAB staining intensity **(B3)** revealed no statistically significant differences between p8-knockout animals and wild type littermates. Results are expressed as mean ± S.D. of islets from 12 animals (6 male, 6 female) per group. Scale bars: 100μm.

To determine whether islet size and islet number contribute to an insulin secretion and content change, glucose-stimulated insulin release and glucagon content was measured *ex-vivo* in islets harvested from p8^−/−^ and wild type mice (Fig 2A1). Both p8^+/+^ and p8 knockout islets revealed glucose-dependent insulin secretion. As is evident in Fig 2A2, p8^−/−^ islets secreted significantly more insulin (5.88±1.9 ng/mg/islet) when exposed to 22 mM glucose compared with islets from control mice (3.15±1.5 ng/mg/islet, p<0.01). In contrast, stimulation with 1.5–22 mM glucose showed no significant differences in insulin levels from islet lysates (Fig 2A3). Thus, islets lacking p8 expression secrete more insulin than wild-type controls when challenged with glucose, suggesting that p8 serves as a negative regulator for insulin secretion. In contrast, insulin content per islet in unstimulated p8-silenced islet lysates did not differ statistically significant (p8^-/-^: 207.8±43.5 ng/mg/islet, p8^+/+^: 216±72.6 ng/mg/islet, p>0.05).

For determination of glucagon staining intensity, pancreatic slides of both p8-knockout and wild type animals were analyzed by means of glucagon DAB staining (Fig 2B1 and 2B2). Quantifications of glucagon staining intensity revealed no statistically significant differences between p8^-/-^ and p8^+/+^ mice. (Fig 2B3). The majority of islets were of low glucagon staining intensity in both groups. Representative images for insulin and glucagon DAB staining and isolated pancreatic islets can be found in Fig 2B1 and 2B2.

### Intraperitoneal insulin tolerance test (ipITT) and glucose tolerance test (ipGTT) in standard and high-fat diet

**Fig 3:**
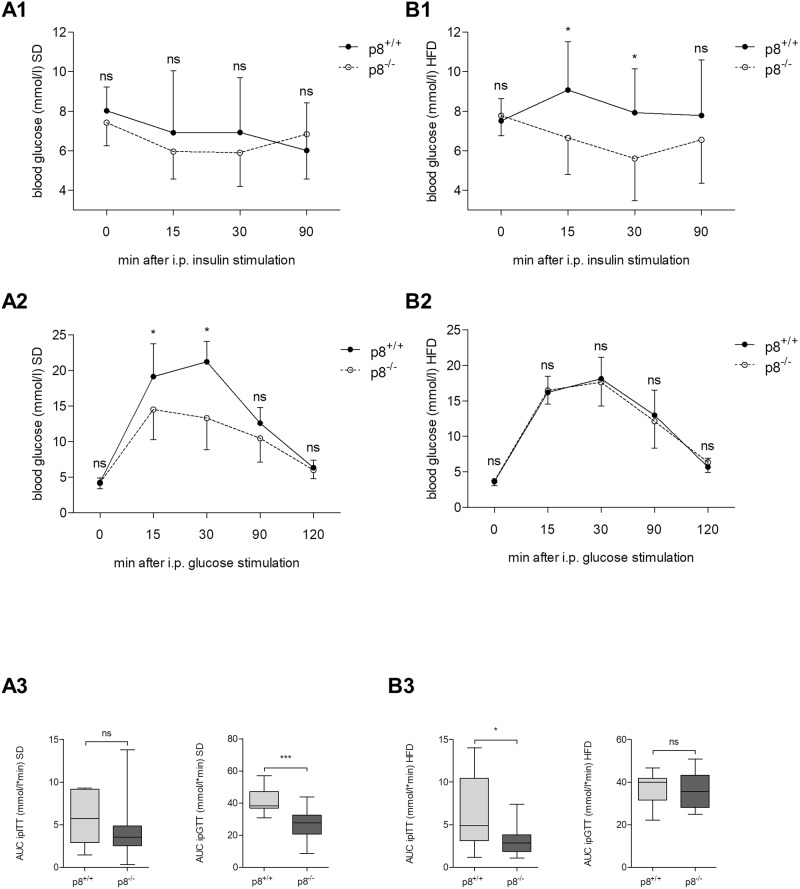
ipITT and ipGTT in standard and high-fat diet. Blood glucose levels were measured in wild type and p8-knockout animals in SD **(A1,A2)** and HFD **(B1,B2)** after i.p. insulin tolerance test (ipITT, **A1,B1**) and glucose tolerance test (ipGTT, **A2,B2**). Unstimulated glucose levels showed no significant alterations. p8^-/-^ mice revealed statistically significant lower glucose levels after ipGTT in SD and after ipITT in HFD. Quantifications of AUC **(A3, B3)** confirmed significant results of tolerance tests. Results are expressed as mean ± S.D. of 12 animals (6 male, 6 female) per group. * P<0.05, *** P<0.001.

To assess the impact of elevated insulin release on glucose disposal, we performed an intraperitoneal glucose tolerance test (ipGTT, 2 g/kg body weight) in p8^−/−^ and control mice under different diet conditions. At 15 and 30 min after administering glucose, there was a significant difference in serum blood glucose level and glucose area under the curve (AUC) between p8^−/−^ (AUC: 26.58±9.6 mmol/l*min) and wild type mice (41.1±7.4 mmol/l*min, p<0.001, Fig 3A2 and 3A3). In contrast, glucose tolerance did not differ in HFD fed mice between p8-knockout (36.4±8.2 mmol/l*min) and wild type rodents (37.5±6.9 mmol/l*min, p>0.05, Fig 3B2 and 3B3). In the ipITT, p8^+/+^ and p8^-/-^ animals showed an early decrease in blood glucose concentrations in response to insulin injection (SD p8^+/+^: 8.0±1.2 mmol/l before vs. 6.0±2.4 mmol/l 90 min after insulin injection, p<0.01, Fig 3A1). Results reveal that p8^−/−^ and control mice had similar blood glucose levels after insulin injection (0.75 U/kg), indicating similar insulin sensitivity under standard chow conditions (AUC 4.2±3.2 mmol/l*min vs. 5.7±2.9 mmol/l*min, p>0.05, Fig 3A1 and 3A3). Under HFD conditions, determination of glucose concentrations upon insulin stimulation showed statistically significant lower levels in p8^-/-^ mice (AUC: 3.15±1.8 mmol/l*min) related to their wild type littermates (6.4±4.1 mmol/l*min, p<0.05, Fig 3B1 and 3B3).

### Hyperinsulinemic-euglycemic clamp

Since tolerance tests do not distinguish between insulin resistance in peripheral tissues and capacity of insulin production by beta cells, we performed hyperinsulinemic-euglycemic clamp experiments at an age of 30 weeks [31].

**Fig 4:**
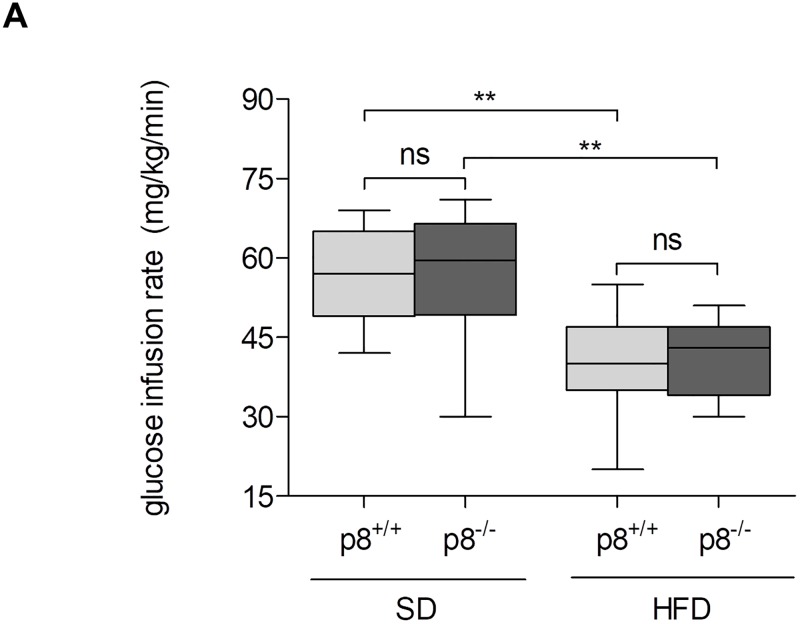
Hyperinsulinemic-euglycemic clamp. **A**, Hyperinsulinemic-euglycemic clamp tests were performed in both groups to analyze insulin resistance. Mice were allowed to maintain steady state after basal infusion of 75 min. Then, insulin perfusion was started at a rate of 40mU/kg/min. Additionally, 20% glucose was infused to maintain blood glucose at euglycemic levels (6.7–7.8 mmol/l) and glucose infusion rate (GIR) was determined. In both p8^+/+^ and p8^-/-^, HFD induced insulin resistance indicated by significantly reduced GIR. However, neither in SD no in HFD, calculation of GIR showed statistically significant differences between p8^-/-^ mice and their wild type littermates **(A)**. Results are expressed as mean ± S.D. of 12 animals (6 male, 6 female) per group. ** P<0.01.

Glucose infusion rate (GIR) was used as an indicator for insulin resistance and therefore determined in p8-knockout and wild type mice fed with SD or HFD. Therefore, a reduced GIR is associated with insulin resistance. Our results clearly showed that insulin resistance was induced by HFD in both wild type and p8-knockout animals (GIR p8^+/+^: 56.3±8.9 vs. 40.6±9.2 mg/kg/min, p<0.01; GIR p8^-/-^: 57.1±11.6 vs. 41.2±7.1 mg/kg/min, p<0.01; Fig 4A). However, our data indicated no significant alterations of GIR between p8-knockout and wild type animals neither in SD nor in HFD fed mice (Fig 4A). In addition, in gender-specific analysis, GIR did not differ between appropriate groups (data not shown).

### Energy expenditure is comparable between p8^-/-^ and control mice

To evaluate the impact of p8 knockout on important metabolic parameters like RER and energy consumption, mice fed with SD and HFD were observed for 72h in metabolic chambers.

**Fig 5:**
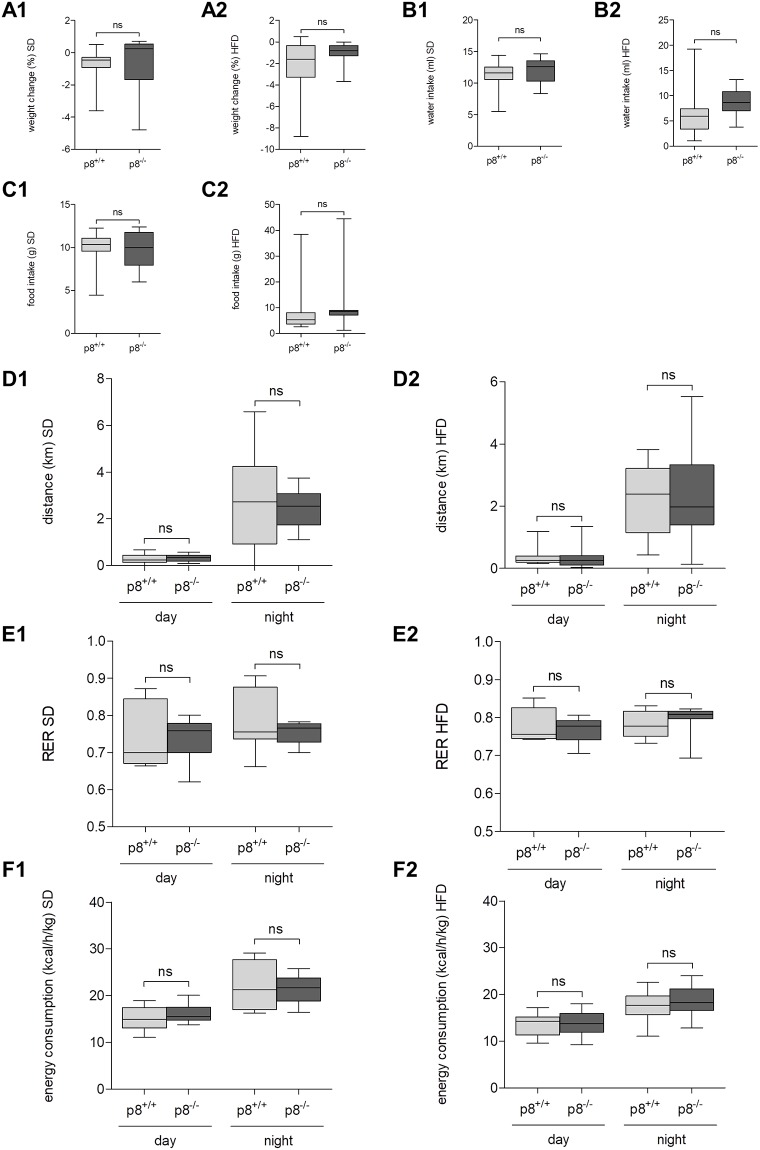
Metabolic chamber analysis. Mice lacking p8 or wild type animals fed with SD or HFD were kept in metabolic chambers for 72h observing weight change **(A1,A2)**, water **(B1,B2)** and food intake **(C1,C2)**, running distance **(D1,D2)**, flow of O_2_ / CO_2_ and temperature. Respiratory exchange rate (RER, **E1,E2**) was calculated by means of O_2_ and CO_2_ flow measurement, and energy consumption rate **(F1,F2)** was estimated from direct calorimetry. No statistically significant alterations between p8-knockout and wild type mice were detected in the aforementioned parameters. Results are expressed as mean ± S.D. of 12 animals (6 male, 6 female) per group. Subgroup analysis of male and female animals also revealed no significant differences.

Between p8^-/-^ and wild type mice, no statistically significant differences were found in weight change, water intake or food intake neither in SD or HFD group (Fig 5A1 and 5C2). p8^-/-^ mice and their wild type littermates covered equal distances in SD group (day: 0.3±0.1 vs. 0.3±0.2 km, p>0.05; night: 2.5±0.9 vs. 2.8±2.2 km, p>0.05, Fig 5D1) and HFD group (day: 0.3±0.3 vs. 0.3±0.3 km, p>0.05; night: 2.3±1.5 vs. 2.2±1.1 km, p>0.05, Fig 5D2). Additionally, analysis of RER showed no significant differences in p8^-/-^ animals and p8^+/+^ mice in SD or in HFD (Fig 5E1 and 5E2). Furthermore, p8-knockout and wild type animals revealed equal energy consumption rate in SD group (day: 16.2±2.0 vs. 15.1±2.6 kcal/h/kg, p>0.05; night: 21.4±2.9 vs. 22.2±5.4 kcal/h/kg, p>0.05, Fig 5F1) and HFD group (day: 13.9±2.5 vs. 13.6±2.4 kcal/h/kg, p>0.05; night: 18.6±3.2 vs. 17.5±3.4 kcal/h/kg, p>0.05, Fig 5F2).

Subgroup analysis of male and female animals also indicated no significant differences between p8-knockout animals and wild type mice (data not shown).

## Discussion

p8 constitutes a stress-activated protein related to high-mobility group box proteins [1] and was originally identified to be overexpressed in sepsis and pancreatitis [5,6]. Prior experiments demonstrate that neither the structure nor function of the exocrine pancreas is altered in p8-knockout mice. Nevertheless, cerulein-induced acute pancreatitis is accompanied by a higher severity of the disease in p8^-/-^ mice compared to wild types [9]. Due to involvement of p8 in cellular mechanisms of oxidative stress, inflammation and proliferation of pancreatic islet cells, p8 depletion may also lead to metabolic dysfunction.

However, the analysis of p8 in transgene mice reveals conflicting data. Whereas complete p8 deficiency results in aggravation of insulin resistance but maintains normoglycemia [20], p8 haplodeficiency protects from hyperinsulinemia and glucose intolerance in HFD-fed animals [21]. Thus, our study analyzed the specific role of p8 in glucose intolerance and insulin resistance by means of insulin or glucose tolerance tests, clamp trials and metabolic chamber analysis in SD and HFD-fed mice.

Recent studies extensively investigated the influence of p8 silencing on insulin levels rather than the analysis of glucagon levels. In addition to these studies, we were able to show that glucagon staining intensity in pancreatic islets from p8^-/-^ mice and their wild type littermates revealed no differences in glucagon levels. On the contrary, determination of insulin levels showed significantly higher insulin secretion but no content change upon glucose stimulation in islets from p8^-/-^ mice. An explanation for higher insulin levels in p8-knockout animals could be a significant elevation of beta cell mass indicated by an increased number and area of pancreatic islet cells. Our results are in line with recent data showing slightly but not significantly elevated fasting insulin levels (about 140 pmol/l) compared to wild type littermates (about 100 pmol/l, p = n.s.), yet indicated elevated insulin levels in p8^-/-^ mice upon glucose stimulation [20]. Nevertheless, absolute insulin concentrations in our data were lower than reported by previous studies. These differences may be the result of different experimental settings. We used isolated pancreatic islets for insulin determinations, contrary to the blood plasma analysis of mice analyzed by prior works [20].

Butler et al. deduces the elevated insulin levels upon glucose stimulation in p8-silenced animals as evidence for insulin resistance as it occurs in diabetes mellitus type 2 [32,33]. On the other hand, our results offered additional explanations for the stimulation of insulin production in p8^-/-^ mice. Since ipITT or ipGTT do not distinguish between insulin resistance in peripheral tissues and beta cell function, we performed hyperinsulinemic-euglycemic clamp experiments. Clamp trials constitute the gold standard test for insulin resistance. The rationale for this test is that high doses of insulin infusion are sufficient to completely suppress hepatic glucose production and that there is no net change in blood glucose concentrations after reaching a steady-state. Under this condition, the rate of glucose infusion (GIR) is equal to the rate of whole-body glucose metabolism. Furthermore, GIR reflects the necessary amount of exogenous glucose to fully compensate for hyperinsulinemia [22]. In our study, insulin resistance was induced by HFD and was indicated by a significantly reduced GIR in HFD animals compared to SD group. Nevertheless, determination of GIR revealed no statistically significant differences between p8-silenced or wild type animals, neither in SD nor in HFD group. In this regard, we could not find any evidence for enhanced insulin resistance in p8^-/-^ mice. Our results of the metabolic chamber tests further support these findings, since no changes in energy consumption rate were detected.

According to our findings, we conclude that the increased beta cell mass and insulin levels upon p8 knockout do not reflect a compensatory mechanism for insulin resistance but rather are a result of enhanced beta cell proliferation and expansion. In this regard, previous studies are in line with our results. It has been observed that fibroblasts isolated from p8^-/-^ mice showed increased cell proliferation [34], and silencing of p8 in pancreatic cell lines also results in the latter [35]. In contrast, p8 overexpression is accompanied by upregulated proliferation of beta cells; however, this was only observed in the presence of glucose [19]. Additionally, p8 overexpression results in reduced beta-cell-related gene expression, insulin content and secretion. In turn, transplanted pancreatic islets overexpressing p8 show induced beta cell growth and insulin production, leading to the assumption of a complex interplay of p8 in beta cell regulation [18,19]. Transcriptional regulation of p8 is well analyzed involving NF-κB and SMAD [36,37], but little is known about molecular control of p8 on protein levels. Therefore, it has been suggested that p8 is regulated in islets by calcium/calmodulin-dependent kinase 4 (CaMK4) [38,39] as well as promotor activities of Ccna2 and Tcf19 [20]. Therefore, the regulation of p8 is complex and not fully understood. The aforementioned enhanced proliferation and activation of beta cells upon p8 induction may be stress-induced in response to glucose.

Our results are further supported by another study evaluating the effect of p8 haplodeficiency in glucose homeostasis. Although it has been assumed that p8^-/+^ mice show improved insulin sensitivity in HFD, only tolerance tests and no clamp experiments were performed. Haplodeficient mice reveal no alterations in insulin levels, beta cell mass or cell proliferation in SD group. The authors deduce that p8^-/+^ mice show reduced expression of stress-induced Hsp70 [21]. These findings are in line with our hypothesis that the contribution of p8 to glucose metabolism and insulin sensitivity is mediated mainly by its property as a regulator for oxidative stress.

Our study has some limitations. We did not evaluate our results from complete p8-knockout mice in haplodeficient animals. Hyperinsulinemic-euglycemic clamp tests of p8^-/+^ mice eventually would further support our findings. Nevertheless, the meaning of haplodeficiency to genetic function and protein expression is often counterintuitive. In addition, we did not analyze effects of p8 knockout on stress-induced pathways in glucose metabolism, but several studies (see above) have investigated this issue. Furthermore, our results of ipITT and ipGTT show some limitations. We found an increase in blood glucose after 90 min in p8 knockout mice. This may be a result of counterregulatory mechanisms to stress response but remains unclear. Furthermore, we found no differences in ipGTT in HFD group between p8^+/+^ and p8^-/-^ animals. Several explanations, including mouse strain, diet and anesthesia might serve as an explanation [23]. Another important issue is the influence of fasting on glucose homeostasis in mice. Typically, mice are fasted overnight (14-18h) or for 5-6h in metabolic studies. Overnight fasting leads to a nearly catabolic state and reduces liver glycogen stores in the mouse. In a consequence, the variability in baseline blood glucose is decreased. Therefore, overnight fasting is recommended when analyzing the glucose utilization as we applied in the protocol of the ipGTTs. Nevertheless, prolonged fasting enhances insulin-stimulated glucose utilization in mice (in contrast to humans). Thus, determination of insulin action should be performed not later than 5-6h fasting or in random-fed animals as it was performed in ipITT in our experiments [23]. In addition, sex can influence metabolic responses of mice, particularly in transgenic phenotypes [40–42]. To reduce the influence of sex on metabolic studies, mice of the same gender or equal numbers of mice from both sex should be used in each experimental group [23], as it was performed in our experimental setting. In addition, subgroup analysis of male and female animals (data not shown) did not reveal statistically significant differences.

To overcome these known limitations of ipGTT and ipITT, we performed clamp trials as the gold standard test for insulin resistance. To avoid the necessity of a large animal number in concordance to the governmental principles for the care and use of animals, we performed ipITT and ipGTT in rodents at the age of 20 wk followed by clamp trials at the age of 30 wk. Thus, this difference in age could be a potential influencer to our results. Nevertheless, we used age-matched wild type and p8-knockout animals to overcome this limitation.

In conclusion, our results show elevated insulin levels upon glucose stimulation and increased beta cell mass in p8-knockout mice as a consequence of induced beta cell proliferation. Therefore, in hyperinsulinemic-euglycemic clamp tests, no evidence for p8 contribution to insulin resistance was observed. Further studies are necessary to evaluate the influence of p8 on glucose homeostasis.

## Supporting information

S1 File (DOCX)

## Abbreviations

AB: antibody
AUC: area under the curve
b.w.: body weight
BSA: bovine serum albumin
CaMK4: calcium/calmodulin-dependent kinase 4
FCS: fetal calf serum
GIR: glucose infusion rate
HFD: high-fat diet
HO-1: heme-oxygenase-1
ipGTT: intraperitoneal glucose tolerance test
ipITT: intraperitoneal insulin tolerance test
IU: international units
NUPR1: Nuclear Protein 1, Transcriptional Regulator gene; p8
P/S: penicillin and streptomycin
PAP I: pancreatitis associated protein I
RER: respiratory exchange rate
RT: room temperature
SD: standard diet
wk: weeks
