# Supplementary material for: p8 deficiency leads to elevated pancreatic beta cell mass but does not contribute to insulin resistance in mice fed with high-fat diet
Source: PLoS One. 2018 Jul 24;13(7):e0201159. doi: 10.1371/journal.pone.0201159 (PMC6057664; doi:10.1371/journal.pone.0201159)
Supplement: S1 File — (DOCX) [file pone.0201159.s001.docx]

**Supplemental Material and methods**

**Genotyping of p8 ^-/-^ mice**

Genomic DNA was isolated from the tail tip by using the DNeasy Kit (Qiagen, Hilden, Germany). Genotyping was performed by PCR (ffw-Primer: GGGTGGTTTAGGGAGGAGACA, rev-Primer: GGAGGTGTGTGAGGTTAGG; program: 94°C for 5min, (94°C for 10s, 60°C for 30s, 72°C for 90s) for 35 cycles, 72°C for 10min).

**Isolation of pancreatic islets**

Pancreatic islets from aged (10-12 wk) p8^-/-^ mice and their wild type littermates (12 per group) were isolated as previously described [17,18]. After anesthesia and laparotomy, the common bile duct was branched off above duodenal entry and cannulated. Immediately, the pancreas was perfused with cold (4°C) collagenase (Roche, Mannheim, Germany) in Hank’s solution (Sigma-Aldrich, Steinheim, Germany). After aseptic harvesting of the pancreas, digestion for 9 min in 37°C water bath in Hank’s solution with collagenase was performed. Subsequently, digestion was stopped by means of cooling down in Hank’s solution on ice, followed by centrifugation at 250 x g for 15s and washing twice in Hank’s solution. Cell suspension was plated on petri dishes, and pancreatic islets were collected under microscopic control. Isolated islets were cultivated in RPMI (Gibco, N.Y., USA) with 10% fetal calf serum (FCS), 1% penicillin/streptomycin (P/S) and 5.5 mM glucose (all from Serva, Heidelberg, Germany) and maintained in culture (37°C, 21% O_2_, 5% CO_2_) for at least 24h prior to the following experiments.

**Insulin determination in pancreatic islets**

For determination of insulin content, 10 pancreatic islets were collected for each measurement. They were resuspended in IRI-buffer (40 mM NaH_2_PO_4_, 100 mM NaCl, 0.3% BSA, ph 7.4, all from Roth, Karlsruhe, Germany) and then lysed with sonic homogenisator (Sonoplus, Bandelin, Berlin, Germany) for 30s at 20% intensity and 4°C. Aliquots were frozen at -20°C until insulin measurements were performed. For detection of insulin secretion from pancreatic islets, we determined insulin levels in the supernatant of the latter.

To analyze both insulin content and secretion, islets were stimulated with 1.5, 5.5, 11 or 22 mM glucose in Gey medium (60 mM NaCl, 2 mM KCl, 13 mM NaHCO_3_, 0.516 mM MgCl_2_x6H_2_O, 0.11 mM KH_2_PO_4_, 0.14 mM MgSO_4_x7H_2_O, 0.31 mM Na_2_HPO_4_x2H_2_O, 0.1% BSA all from Roth, Karlsruhe, Germany) for 2h in an incubator (37°C, 21% O_2_, 5% CO_2_). After stimulation, either 100 µl of supernatant was collected for analysis of insulin secretion, or islets were resuspended in 500 µl IRI-buffer followed by the above mentioned homogenization. IRI buffer with 0.1% BSA without glucose was used as a control.

Insulin levels were measured by using an ultra-sensitive mouse ELISA Kit (Chrystal Chem Inc., Downers Grove, IL, USA) following instructions from the manufacturer.

**Conflicts of Interest:**

The founding sponsors had no role in the design of the study, in the collection, analyses, or interpretation of data, in the writing of the manuscript or in the decision to publish the results.

**Acknowledgements**

The authors declare that they have no conflict of interest. We would like to thank Tiffany Schaumburg for her critical language editing.
